# Changes in Quality of Life Among Glaucoma Patients Following Six Months of Niacinamide Supplementation

**DOI:** 10.3390/nu17172775

**Published:** 2025-08-27

**Authors:** Constantin Alin Nicola, Maria Cristina Marinescu, Anne Marie Firan, Georgica Tartea, Mihaela Simona Naidin, Radu Constantin Ciuluvica, Marina Daniela Dimulescu, Nicoleta Mirela Voicu, Carmen Marinela Mihailescu, Andreea-Daniela Meca, Maria Bogdan, Adina Turcu-Stiolica

**Affiliations:** 1 Doctoral School, University of Medicine and Pharmacy of Craiova, 200349 Craiova, Romania; 2 Discipline of Medical Physiology, Faculty of Medicine, Carol Davila University of Medicine and Pharmacy, 020021 Bucharest, Romania; 3 Barnsley Hospital, Barnsley Hospital NHS Foundation Trust, Barnsley S75 2EP, UK; 4 Department of Physiology, Faculty of Medicine, University of Medicine and Pharmacy of Craiova, 200349 Craiova, Romania; 5 Department of Pharmaceutical Marketing and Management, Faculty of Pharmacy, University of Medicine and Pharmacy of Craiova, 200349 Craiova, Romania; 6 Discipline of Anatomy, Faculty of Dentistry, Carol Davila University of Medicine and Pharmacy, 020021 Bucharest, Romania; 7 Discipline of Clinical Pharmacology, Faculty of Medicine, Carol Davila University of Medicine and Pharmacy, 020021 Bucharest, Romania; 8 Discipline of Pharmacology, Faculty of Pharmacy, University of Medicine and Pharmacy of Craiova, 200349 Craiova, Romania

**Keywords:** glaucoma, vitamin B3, niacin, niacinamide, quality of life

## Abstract

**Background/Objectives:** Glaucoma is the primary cause of irreversible blindness worldwide, with enormous impact on quality of life and activities of daily living. Since one pathogenic mechanism of glaucoma is mitochondrial dysfunction at the retinal ganglion cell level, niacin has been proposed as an adjuvant treatment, with encouraging results. The objective of this prospective, non-randomized, single-arm clinical trial was to investigate the effect of oral supplementation with niacin on the quality of life of a cohort of glaucoma patients in Romania. **Methods:** Fifty-eight patients diagnosed with primary open angle glaucoma, under topical hypotensive treatment, were evaluated before and after a 6-month period of daily administration of 500 mg of oral niacinamide. Evaluation involved a complete ophthalmological exam and QoL quantification using the Glaucoma Quality of Life-15 (GQL-15) Questionnaire. **Results:** We found strong evidence that niacin supplementation for 6 months led to a statistically significant improvement in QoL scores among glaucoma patients (mean difference = −2.10, 95% CI: [−2.89, −1.32], *p* < 0.0001), including central and near vision (mean difference = −2.16, 95% CI: [−3.91, −0.4], *p* = 0.017), peripheral vision (mean difference = −2.66, 95% CI: [−0.23, −0.08], *p* < 0.001), and the glare and dark adaptation (mean difference = −5.24, 95% CI: [−0.33, −0.14], *p* < 0.001). In addition, B3 treatment resulted in a significant reduction in intraocular pressure in both eyes over 6 months (mean difference = 0.53, 95% CI: [0.21, 0.86] in the left eye and mean difference = 0.36, 95% CI: [0.04, 0.68] in the right eye), indicating potential clinical benefits. **Conclusions:** The observed GQL-15 score reductions suggest that B3 may be of benefit in glaucoma management. Further research with larger sample sizes and placebo-controlled designs is needed to confirm B3 potential impact on disease progression and quality of life. Trial Registration at clinicaltrials.gov: NCT07007260.

## 1. Introduction

Glaucoma is a serious eye disease, with an estimated prevalence of 76 million in 2020 and an upwards trend towards 112 million by 2040. Of particular concern, glaucoma is the leading cause of irreversible blindness worldwide [1,2]. Approximately 5.7 million people suffer from visual impairment due to glaucoma, and around 3.1 million are blind due to the evolution of the disease [3]. Quality of life (QoL) is defined by the World Health Organisation as the subjective perception of each person’s reality, in the given cultural context, and the subjective perception of each individual’s well-being [4,5]. Through their impact on activities of daily living, ocular disease and glaucoma in particular significantly reduce quality of life. A systematic review involving vision impairment reveals a lower quality of life, both general vision-related and health-related: people with vision impairment are 2.61 times more likely to have a poorer score regarding mental health, fatigue or psychosocial functioning [6]. Specifically for glaucoma, a cross-sectional study reveals that 11.2% of patients suffer from and 26.0% from depression [7]. An important measure of the impact of the disease, the number of years of healthy life lost to disability (DALY—disability-adjusted life years), is also reported for glaucoma by the Global Burden of Diseases study: the total burden was 748,307.7 DALYs in 2019, although this was unevenly distributed, with a higher burden of disease in low socioeconomic level regions and aging populations [8].

Functionally, glaucoma affects quality of daily life by interfering with peripheral vision (and later in the progression, central vision), color perception, contrast sensitivity and by disrupting the light–dark adaptation [9]. Moreover, glaucoma may bring a psychological burden (fear of blindness), the burden of potential medical and surgical side effects, and the financial aspects of regular follow-up, interventions and loss of workdays [5].

As glaucoma is a progressive disease of the optic nerve, it is considered a neurodegenerative pathology and therefore attempts have been made to address the axonal degeneration directly. Currently, the only modifiable risk factor and treatment target is elevated intraocular pressure (IOP) [3]. The pathophysiology of the disease is complex, but the role of mitochondrial dysfunction is increasingly recognised, and several molecules are investigated that may improve this aspect, such as niacinamide. Also called nicotinamide, niacinamide is a precursor of NAD (nicotinamide adenine dinucleotide), an enzyme involved in redox reactions, and therefore in metabolic pathways (glycolysis, fatty acid oxidation, oxidative phosphorylation) and post-translational processes of DNA repair and stress response. In addition, it is known that disturbances in the NAD metabolism are involved in axonal degeneration—in particular, the depletion of NAD deposits [10,11]. As the age of the patients increases (an additional glaucoma risk factor), the homeostasis of NAD reserves in the retinal cells is disturbed, and the ganglion cells become more vulnerable to IOP-related stress [12]. Animal model studies suggest that oral supplementation of niacinamide may protect against glaucomatous degeneration, and studies involving human patients are underway, with some even suggesting a lower dietary intake of niacin in glaucoma patients and a 33–37% lower likelihood of glaucoma in the high intake group [13,14]. In the current study, we investigated the effect of B3 supplementation on the important aspect of glaucoma—the impact on the quality of daily life.

## 2. Materials and Methods

### 2.1. Recruitment of Patients

This study has a non-randomized interventional methodology. The cohort study was formed by applying inclusion and exclusion criteria to all patients who consecutively presented to a private Ophthalmology practice in Bucharest, Romania. The study was performed between March 2023 and March 2024. The study was conducted in accordance with the Declaration of Helsinki and approved by the Ethics Committee of the University of Medicine and Pharmacy of Craiova, Romania (no. 50/10.02.2023). Informed consent was obtained from all subjects involved in the study. The study is registered at ClinicalTrials.gov, with ID NCT07007260.

The inclusion criteria were the prior diagnosis of primary open-angle glaucoma (POAG), based on slit-lamp examination, dilated fundoscopy, ocular tonometry, visual field examination and optical coherence tomography (OCT) examination.

The exclusion criteria were the presence of ocular pathology, other than glaucoma (advanced cataract, degenerative myopia, keratoconus, amblyopia, vitreoretinal pathology, ocular inflammation or significant sequelae). Furthermore, patients were excluded in the absence of testing compliance (high positive and negative errors on visual field examination), if the patient was pregnant, or if the glaucoma was very advanced (Bascom Palmer stage 5—blindness, with no visual field in the worst eye) [15].

The patients included in the study had undergone a complete ophthalmological examination, including visual acuity at distance (quantified under the logMAR scale), gonioscopy, slit lamp examination of the anterior and posterior ocular segments and non-contact tonometry (I-Care tonometer). Additionally, patients completed the Glaucoma Quality of Life-15 (GQL-15) Questionnaire, developed by Nelson et al. [16]. Also reported are the duration of the disease and the number of antiglaucoma molecules administered daily. Glaucoma cases were classified according to the Bascom Palmer GSS criteria for perimetric MD: stage 1 (early glaucoma): MD over −6.00 dB; stage 2 (moderate glaucoma): MD over −12.00 dB, stage 3 (advanced glaucoma): MD over −20.00 dB and stage 4 (severe glaucoma): MD −20.01 dB or worse [15].

### 2.2. Glaucoma Quality of Life-15 Questionnaire

Patients’ QoL was assessed in correlation with age, gender, visual field (VF) defect, severity of glaucoma, duration, and the number of antiglaucoma medications they were taking. The QoL was evaluated using translated Glaucoma Quality of Life-15 (GQL-15) [16,17], a glaucoma-specific questionnaire with 15 vision-related items, at baseline (T0) and after six months of daily niacinamide supplementation (T6). A total score of QoL and four subscales were evaluated: central and near vision (CNV) (items 1 and 15), peripheral vision (PV) (items 4, 8, 9, 11, 12, and 13), glare and dark adaptation (GDA) (items 2, 3, 5, 6, 7, and 14), and outdoor mobility (OM) (item 10). Every item is coded on a five-point scale (1 meaning no difficulty, 2 a little bit of difficulty, 3 some difficulty, 4 quite a lot of difficulty, and 5 meaning severe difficulty, while 0 is marked if the participant does not perform the activity as a result of non-visual cause). Total score is derived by summing all item-level response scores, where the highest score is 75 and the lowest is 15. Higher GQL-15 scores reveal a lower QOL. Subscale scores are associated with binocular VF loss and are derived by coding the item-level responses on a numerical interval scale ranging from 0 (no difficulty) to 100 (severe difficulty). Subscale scores are the average of the sum of scores generated for the item-level subscale responses. Higher subscale scores indicate greater difficulty in performing vision-related activities and lower QOL. In our study, the GQL-15 questionnaire was completed by the patients in the presence of a physician.

All patients underwent these measurements at the beginning of the study (T0). Subsequently, they were administered niacinamide, 500 mg capsules, once daily, for 6 months. After the treatment period, they repeated all ophthalmic examinations (T6). No modifications in the topical or systemic glaucoma treatment were performed throughout the study.

### 2.3. Statistical Analysis

In this article, we studied categorical and numerical data. For categorical variables, the absolute and relative frequencies were provided. For numerical variables, the average and the standard deviation were calculated.

In order to determine if any significant differences occurred between T0 and T6, a T-test for paired samples was performed when data distribution was normal or Wilcoxon test when data distribution was not normal. Kolmogorov–Smirnov normality tests were also performed. The *p* value of 0.05 was chosen as a representative of statistical significance. All statistical calculations were performed using GraphPad PRISM (version 10.5.0 for Windows) and R packages (version 4.1).

The heatmap was used to visually illustrate the correlations between variables (Pearson’s correlation coefficients, Pearson’s r, or Spearman correlation coefficients (rho) were calculated). A weak correlation is represented by a coefficient between 0.3 and −0.3, a moderate correlation between 0.3 and 0.5 or between −0.3 and −0.5, and a strong correlation over 0.5 or under −0.5.

Post hoc power analysis was conducted using G*Power (version 3.1.9.7) and the conventional 80% threshold was used.

The total GQL-15 score represented the primary outcome, while the subscale scores of CNV, PV, GDA and OM, along with the IOP levels, were secondary outcomes.

## 3. Results

### 3.1. Patients’ Characteristics

**Figure 1:**
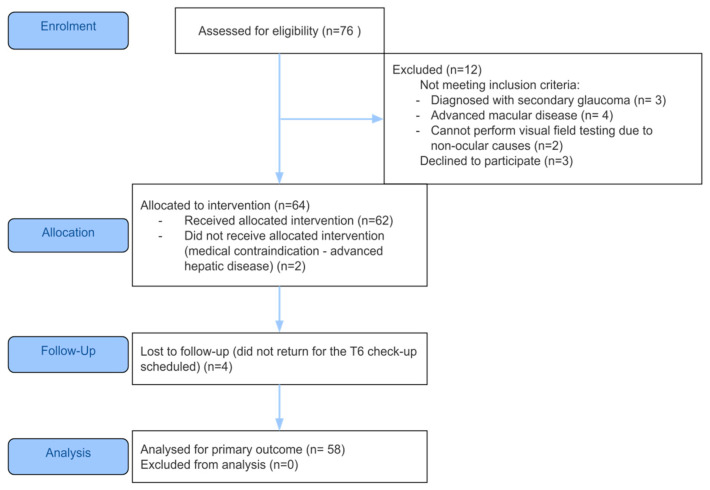
Flow diagram of the inclusion process of the patients in the cohort study.

Fifty-eight patients with glaucoma that met the eligibility criteria were enrolled, as shown in Figure 1. The mean ± standard deviation age of the patients was 66.72 ± 11.75 years and most patients were women (45, 77.6%). All patients had POAG, stratified by disease severity: early defect—30 patients (52%); moderate defect—11 patients (19%); advanced defect—7 patients (12%); severe glaucoma group—7 patients (12%).

### 3.2. Patients’ Quality of Life at Baseline

Patients with more severe visual field defects reported increasing difficulty across most GQL-15 items. Item 1 (Reading newspapers) showed a gradual increase in difficulty with disease progression, from a mean of 1.90 in early stage to 2.43 and 2.29 in advanced and severe stages, respectively. Night vision-related items, such as “Seeing at night” (item 3) and “Walking after dark” (item 2), also demonstrated higher difficulty in more severe stages. For instance, scores for “Seeing at night” (item 3) rose from 2.23 (early) to 2.57 (severe). Mobility-related tasks, including “Walking on uneven ground” (item 4), “Walking on stairs” (item 11), and “Crossing the road” (item 10), exhibited clear upward trends, reflecting functional impairment with glaucoma severity.

Item 15 (“Recognizing faces”)—a task typically associated with central vision—remained relatively low across groups but showed a slight increase in difficulty in severe glaucoma (1.86), suggesting some central vision compromise in advanced disease.

The mean scores across the four GQL-15 subscales further confirmed a pattern of worsening QoL with increasing disease severity. CNV scores increased from 15.42 ± 15.29 in early-stage glaucoma to 26.79 ± 36.39 in severe cases. PV difficulty similarly increased, with mean scores rising from 14.31 ± 13.16 (early) to 26.78 ± 26.44 (advanced). The GDA subscale showed relatively high scores across all groups, with the highest burden reported in moderate to advanced stages (33.33 to 35.72), indicating that light adaptation problems are prevalent even in earlier disease. OM scores were lowest in early-stage patients (6.67 ± 13.02) and substantially higher in advanced and severe groups (both 25.0), suggesting increasing mobility impairment as glaucoma progresses.

The GQL-15 score and its four subscales (CNV, PV, GDA, OM) were compared across the four glaucoma stages. Results indicated no statistically significant differences between glaucoma stages for any of them: for QoL score (F = 0.368, *p* = 0.777), CNV (F = 0.503, *p* = 0.686), PV (F = 0.547, *p* = 0.658), GDA (F = 0.129, *p* = 0.942), and OM (F = 1.188, *p* = 0.350).

Although the rise in total score was modest, the increasing variability (as seen in the growing standard deviations in Table 1) suggests greater heterogeneity in perceived QoL as disease progresses.

### 3.3. GQL-15 Score and Subscale Analysis

**Figure 2:**
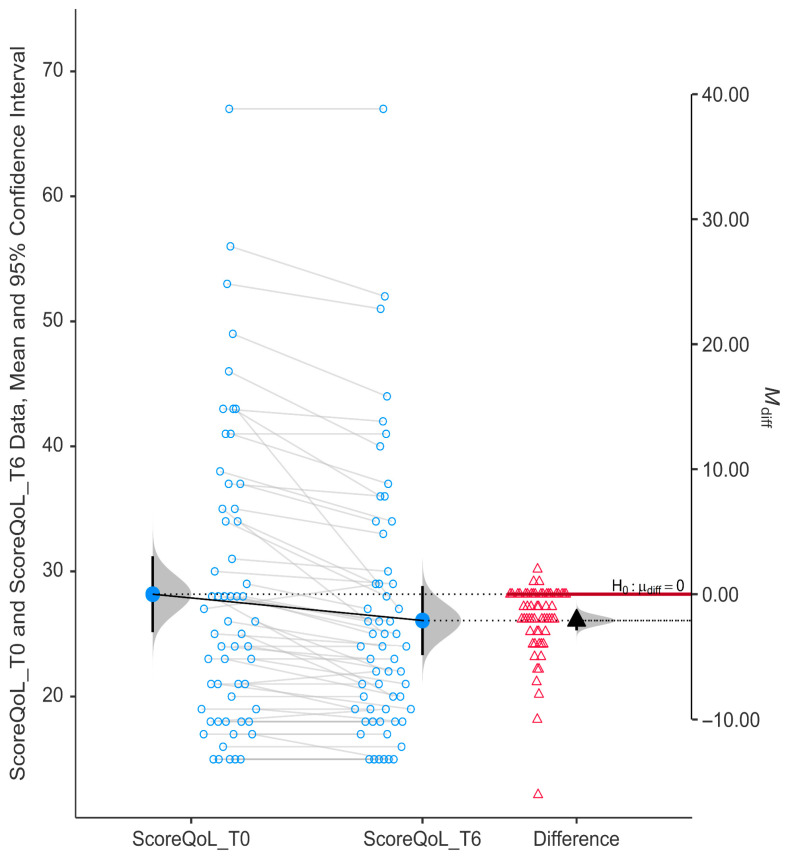
Change in score of QoL using GQL-15 (Glaucoma Quality of Life-15), where higher scores indicate worse QoL. Paired data (blue open circles) for each participant before (T0) and after treatment (T6). The black solid triangle represents the mean of the red open triangles (the difference in the score of QoL for every patient).

We found strong evidence that niacinamide supplementation over 6 months led to a statistically significant improvement in QoL scores among glaucoma patients (*p* < 0.0001). The drop in GQL-15 scores further supports a marked improvement in vision-related quality of life after niacinamide supplementation (Figure 2). Mean QoL score decreased from T0 (28.17 ± 11.53) to T6 (26.07 ± 10.51), indicating improvement in QoL. The mean difference was negative (−2.10) and the 95% confidence interval did not cross zero (95% CI, −2.89 to −1.32).

**Figure 3:**
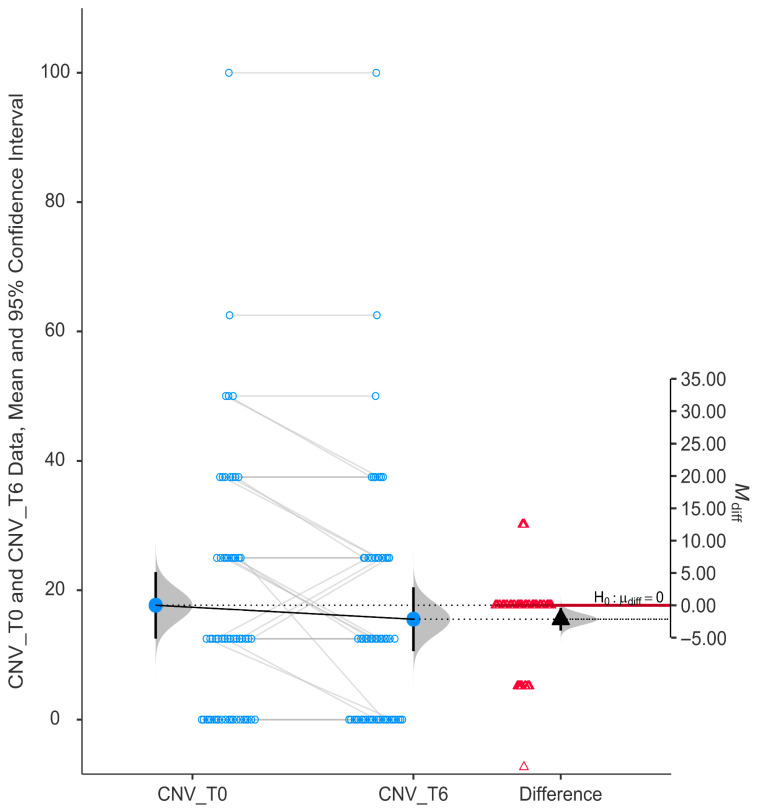
Change in score of central and near vision (CNV) using GQL-15.

The subscale evaluating central and near vision (CNV) showed a statistically significant improvement after six months of niacinamide supplementation (*p* = 0.017). The mean CNV score at T0 (15.52 ± 18.78) was higher than at T6 (17.67 ± 19.59), and the mean difference (M_diff) was negative (−2.16). The 95% confidence interval for the difference did not cross zero (−3.91 to −0.399), as shown in Figure 3.

**Figure 4:**
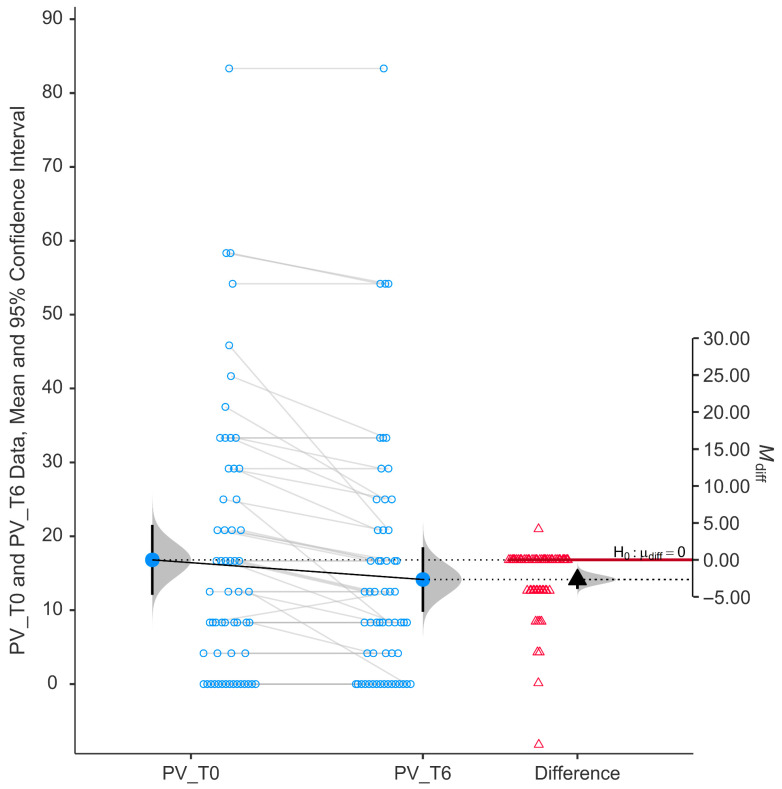
Change in score of peripheral vision (PV) using GQL-15.

Peripheral vision (PV) scores showed a statistically significant improvement following six months of niacinamide supplementation (*p* < 0.001). As shown in Figure 4, individual paired data indicate a consistent reduction in self-reported difficulty, with most data points trending downward from baseline (mean ± SD, 16.81 ± 18.02) to follow-up (mean ± SD, 14.15 ± 16.61).

The histogram of the differences (right side of the figure) shows a distribution of changes centered below zero, with a mean difference (M_diff) that is negative (−2.66) and a 95% confidence interval not overlapping zero (from −0.231 to −0.076), supporting a statistically significant improvement (*p* < 0.001).

The group mean peripheral vision score decreased from baseline to T6, indicating less difficulty in performing tasks requiring peripheral vision, such as navigating through crowds or detecting objects outside the central visual field.

**Figure 5:**
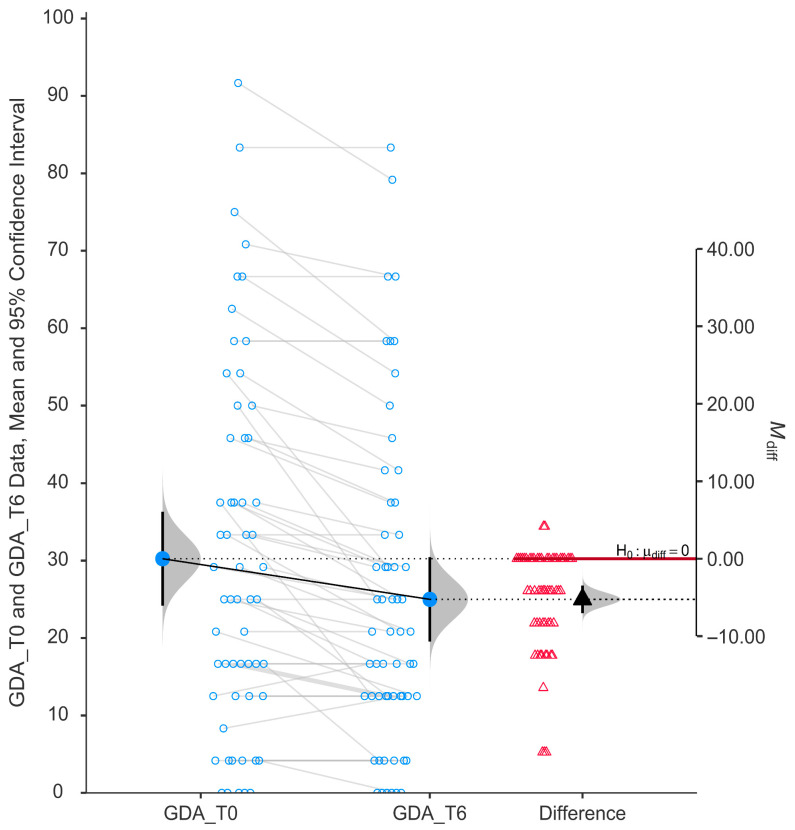
Change in score of glare and dark adaptation (GDA) using GQL-15.

The glare and dark adaptation (GDA) subscale demonstrated a statistically significant improvement after six months of niacinamide supplementation (*p* < 0.001). As illustrated in Figure 5, individual participant data show a consistent trend of reduced scores from baseline (mean ± SD, 30.24 ± 23.12) to post-treatment (mean ± SD, 25.00 ± 20.75), indicating an overall decrease in self-reported difficulty with tasks involving glare sensitivity and dark adaptation.

The paired data plot reveals a mean reduction in GDA scores, and the violin plot of the difference scores confirms that most changes are clustered below zero. The mean difference (−5.24) was statistically significantly negative, with the 95% confidence interval not crossing zero (−0.33 to −0.14).

**Figure 6:**
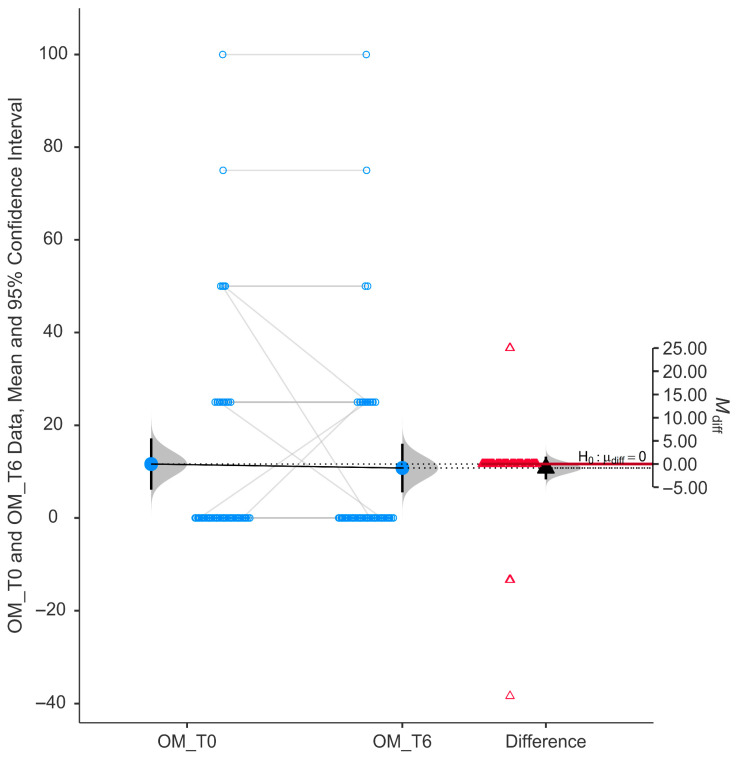
Change in score of outdoor mobility (OM) using GQL-15.

The Outdoor Mobility (OM) subscale did not show a statistically significant difference following six months of niacinamide supplementation. As depicted in Figure 6, the paired data reveal a relatively symmetrical distribution around the zero-difference line, with minimal change in mean scores between baseline (11.64 ± 21.06) and follow-up (10.78 ± 19.93).

The violin plot of difference scores displays a tight distribution centered near zero, and the mean difference (−0.862) was close to zero with a 95% confidence interval that crosses the null line (H_0_: μ_diff = 0, 95% CI from −0.16 to 0.076). These findings indicate no significant improvement in this specific domain of quality of life.

**Figure 7:**
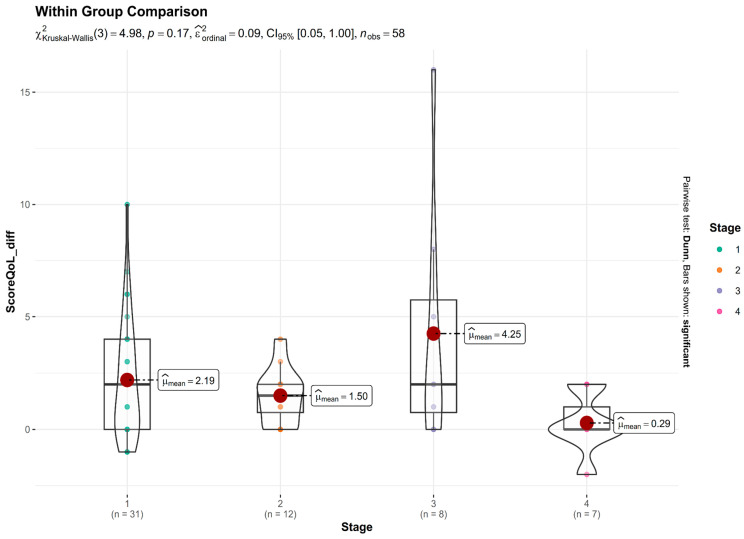
The distribution of change in QoL scores (difference between baseline and post-treatment) across four glaucoma severity stages (1: Early stage, 2: Moderate, 3: Advanced, 4: Severe). A Kruskal–Wallis test was conducted to assess whether the degree of improvement differed significantly among the stages.

Figure 7 presents the distribution of change in QoL scores (difference between baseline and post-treatment) across four glaucoma severity stages. A Kruskal–Wallis test was conducted to assess whether the degree of improvement differed significantly among the stages. The test revealed no statistically significant difference: χ^2^(3) = 4.98, *p* = 0.17. While statistical significance was not reached, there was a trend suggesting greater improvements in Stage 3 (Advanced) glaucoma patients, who showed the largest mean reduction in QoL burden. In contrast, Stage 4 (Severe) glaucoma patients experienced minimal benefit, with a mean change close to zero. Patients with early and moderate disease stages also reported improvements, though the magnitude was smaller than that observed in advanced cases.

### 3.4. Correlations Between Changes in Intraocular Pressure, Clinical Parameters and Quality of Life

**Figure 8:**
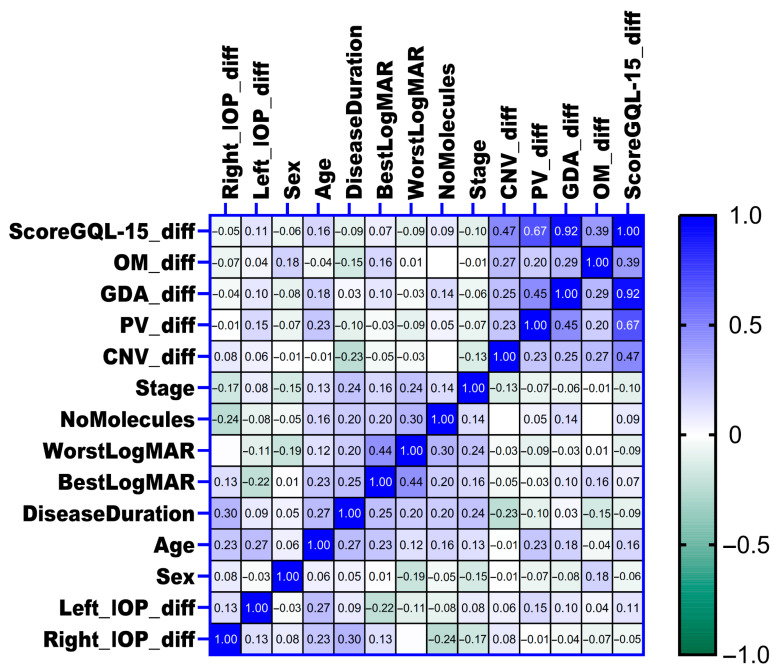
The heatmap illustrates the correlations between the changes in intraocular pressure (Right_IOP_diff for right eye and Left_IOP_diff for left eye) and the different clinical variables and quality of life indicators. Each cell in the heatmap represents the strength of correlation (rho value), with colors ranging from green (negative correlation) through white (no correlation) to blue (positive correlation). Stronger correlations are depicted with more intense coloration. IOP_diff, intraocular pressure difference; NoMolecules, number of INNs (International Nonproprietary Names).

The associations between the changes in intraocular pressure (IOP) in both eyes and various clinical parameters, including glaucoma stage, number of concurrent glaucoma medications (number of molecules), disease duration, age, sex, visual acuity measured by worst and best LogMAR at baseline, and changes in quality of life as assessed by the GQL-15 score and its subscales, were examined using Spearman correlation analysis. Overall, no significant correlations were identified between changes in IOP and most clinical variables or quality of life measures (as shown in Figure 8). However, a positive correlation was detected between changes in right eye intraocular pressure (Right_IOP_diff) and disease duration (rho = 0.3, *p* = 0.029), suggesting that longer disease duration was moderately associated with increased intraocular pressure enhancement in the right eye. Similarly, a positive correlation was identified between age and changes in left eye intraocular pressure (Left_IOP_diff) (rho = 0.3, *p* = 0.045), indicating that older age was moderately associated with increased intraocular pressure enhancement in the left eye.

### 3.5. Intraocular Pressure Change Analysis

**Figure 9:**
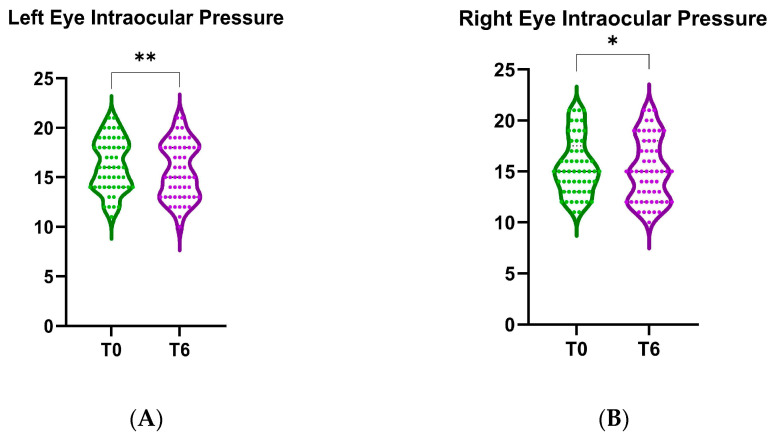
Effect of niacinamide supplementation on eye intraocular pressure over 6 months. (**A**) Left eye. (**B**) Right eye. *, *p* < 0.05; **, *p* < 0.01.

Violin plots comparing the baseline (T0) and 6-month follow-up (T6) intraocular pressure after B3 treatment are presented in Figure 9. We found a statistically significant decrease in IOP for the left eye (mean difference of the change in IOP = 0.53, 95% CI: [0.21, 0.86], *p* < 0.001, as shown in Figure 9A). Similarly, the right eye also exhibited a significant reduction in IOP after 6 months of treatment (mean difference of the change in IOP = 0.36, 95% CI: [0.04–0.68], *p* < 0.05, as shown in Figure 9B).

A post hoc power analysis was conducted for the Wilcoxon signed-rank test (matched pairs) and the achieved power was 93.4%, indicating a very high probability of correctly rejecting the null hypothesis if the true effect exists, far exceeding the conventional 80% threshold for sufficient power. This suggests that the study including 58 patients was well powered to detect the observed effect size, with the sample size being more than adequate.

## 4. Discussion

The Glaucoma Quality of Life-15 (GQL-15) Questionnaire was published in 2003 by Nelson and colleagues, with the aim of correlating objective visual loss in glaucoma, primarily visual field defects, with perceived quality of life. It consists of 15 questions that mainly assess daily activities, which involve dark adaptation, peripheral vision or glare. The questionnaire also correlates well with perimetric variables and has high retest reliability [16]. It has been independently assessed and confirmed the correlation with objective visual loss and the significant difference to the visual function of normal individuals [15].

The mean GQL-15 score in our study was 28.17 ± 11.53 before B3 supplementation (ranging from 26.97 ± 9.21 in early disease to 31.71 ± 18.28 in severe disease). This is comparable to other populations to which the questionnaire was translated and adapted for—the average value was 26.00 ± 10.84 in an Indian population [5], 39.50 ± 16.76 in a Moroccan Arabic population [18], 20.68 ± 7.31 in a Serbian population [19] and 29.3 ± 7.31 in a Spanish-speaking population [20].

Regarding the subscales reported by the GQL-15 questionnaire, peripheral vision is the first to be affected by glaucoma, with central vision also being affected in advanced cases. Other aspects of vision are also affected (color perception and contrast, along with altered light-dark adaptation)—which all affect outside orientation and activities of daily living [9].

This single-center study assessed the impact of six months of oral niacinamide supplementation on the quality of life (QoL) of glaucoma patients using the GQL-15 questionnaire. The results show a statistically significant improvement in overall QoL, as well as in multiple vision-specific subdomains: central and near vision, glare and dark adaptation and peripheral vision. While statistically significant, the magnitude of change appears modest, highlighting the importance of interpreting these improvements in the context of clinical relevance. Further studies should assess whether these improvements persist over longer follow-up and whether objective measures (e.g., reading speed, visual acuity) correlate with the observed self-reported gains. Moreover, more research is needed into the objective measures of ocular physiology (visual evoked potentials, visual field testing) in order to correlate these subjective benefits of niacinamide supplementation.

Our findings support the hypothesis that niacinamide supplementation is associated with improved CNV function, as perceived by patients. CNV tasks—such as reading, recognizing faces, and using mobile devices—are essential for daily functioning, especially in older adults with glaucoma [21]. Nearly 50% of ganglion cells are centrally located in the retina and over 90% of the visual cortex processes information originating from the central 10° of the visual field [22]. As the role of niacinamide is increasingly evident in the energetic metabolism of the ganglion cell [12], our finding is consistent with the hypothesized neuroprotective effect of niacinamide on retinal ganglion cells.

PV subscale scores also showed statistically significant improvement post-treatment. Glaucoma typically affects peripheral vision first, traditionally described as evolving into “tunnel vision” [23]; therefore, even small improvements or stabilization in this domain are clinically valuable. Patients reported reduced difficulty with activities that require peripheral awareness, such as walking through crowds or detecting objects in the periphery. In the literature, more than a third of patients describe difficulty seeing objects which are placed off to both the right and left side, and more than a quarter report difficulties in seeing things either to the right or to the left [23]. Trials in human subjects suggest some objective benefits of niacinamide (in combination with pyruvate) in visual field improvements (odds ratio of 3.20 of improving visual field points tested, in comparison to the control group) [24], therefore corroborating the perceived improved quality of peripheral vision perceived by our group in the present study.

The most pronounced subscale improvement was observed in the GDA domain. Patients experienced significantly less difficulty adjusting to changes in lighting conditions or coping with glare. Because glare sensitivity and poor dark adaptation are commonly reported by glaucoma patients, especially in low-light environments, this result is highly relevant to daily function and safety (e.g., driving at night or walking outdoors at dusk). Studies report that, after a sudden increase or decrease in luminance, adaptation curves of glaucoma patients are altered compared to healthy controls [25], and, particularly in severe cases, the presence of glare significantly decreases the visual acuity of glaucoma patients [26]. A systematic review confirms that GDA is also the most problematic subscale of the GQL-15 test in many other populations [27]. Mechanisms for GDA difficulties in glaucoma may include a selective glaucomatous damage to magnocellular ganglion cells, responsible for contrast under mesopic and scotopic conditions [27]. These improvements may be due to enhanced retinal resilience and energy metabolism, as niacinamide boosts NAD+ levels [28]

In contrast, the OM subscale showed no statistically significant change after niacinamide supplementation. This item, based on a single question, may not be sensitive and is likely influenced by non-visual factors such as musculoskeletal limitations, general health status, or environmental factors [19,29]. In addition, many patients reported low difficulty in this domain at baseline, possibly limiting the potential for observed improvement.

These improvements are consistent with emerging evidence suggesting that niacinamide may enhance mitochondrial function and support retinal ganglion cell health, potentially delaying or stabilizing functional loss in glaucoma [14]. However, the present research suggests an additional mechanism of action of niacinamide: after 6 months of B3 supplementation, the intraocular pressure was modestly yet significantly lower. Such a reduction was not observed in other studies [28]; however, preclinical studies have proven that niacinamide also acts upon the mitochondria and the energy metabolism of other ocular tissues, such as the trabecular meshwork, preventing abnormal deposition of extracellular matrix and a decrease in aqueous outflow, therefore indirectly lowering the IOP [30,31]. Further research is needed to decipher both the mechanism and potential clinical benefit of niacinamide’s impact on IOP.

Our study shows that the differences between groups did not reach statistical significance, likely due to small sample sizes (especially in the more advanced stages), meaning the trend is clinically informative. Advanced-stage (Stage 3) patients experienced the largest improvements, suggesting that niacinamide may offer the most perceptible benefit in patients at a functionally vulnerable but not end-stage phase of glaucoma. This aligns with prior research indicating that retinal ganglion cells in moderate-to-advanced stages may still be viable but metabolically stressed—making them more responsive to metabolic support such as NAD+ augmentation [32].

In contrast, severe-stage (Stage 4) patients showed minimal changes, possibly due to extensive, irreversible structural damage, limiting the potential for functional recovery even with supplementation [33]. Early-stage patients may already have relatively preserved QoL, which limits the magnitude of measurable improvement, while moderate cases showed modest benefit. These findings suggest a possible “therapeutic window” in moderate to advanced glaucoma, where niacinamide supplementation might be most effective in improving subjective QoL.

Further studies with larger subgroup sizes and longer follow-up are warranted to confirm these trends and determine the optimal timing for metabolic interventions in glaucoma care.

The study presents several limitations—the supplementation was not blinded to the patients and they were informed they would be administered niacinamide; therefore, there is a possible role for the placebo effect. However, the consistent improvements observed across several GQL-15 subscales, together with the significant reductions in IOP—an objective parameter less susceptible to placebo influence—support the likelihood of a true treatment effect. Nonetheless, future randomized, double-blind, placebo-controlled trials are necessary to definitively confirm the causal impact of niacinamide supplementation on both patient-reported and objective outcomes.

Despite its widespread use, the GQL-15 questionnaire has several limitations. It primarily assesses visual function, neglecting psychological, social, and treatment-related factors that significantly influence quality of life in glaucoma patients [34]. We considered the GQL-15 questionnaire offered several advantages in evaluating glaucoma-related quality of life. It specifically focuses on visual function and daily activities that impact glaucoma, providing clinically relevant insights. Additionally, the GQL-15 has been widely validated across diverse populations and translated into multiple languages, supporting its international applicability and comparability across studies. Its well-established status in glaucoma research facilitates comparison and interpretation of findings within existing literature [5,18,19,20].

Although the overall study was well powered (93.4% for paired comparisons) for the IOP change analysis, subgroup analyses by glaucoma stage were limited by small sample sizes, particularly in the advanced and severe categories. As a result, the lack of statistically significant differences between stages should be interpreted with caution, and firm conclusions about stage-specific efficacy cannot be drawn. Larger studies with stratified enrollment are needed to clarify whether certain stages of glaucoma benefit more from niacinamide supplementation. We also observed variability in treatment response, with some patients showing marked improvements in GQL-15 scores and others showing little change. Due to the limited sample size, we did not perform responder versus non-responder analyses or multivariable regression, but such approaches in larger cohorts could help identify patient subgroups most likely to benefit from niacinamide supplementation.

Another limitation is that niacinamide (or metabolite) blood levels were not measured, which would have provided objective confirmation of supplementation, adherence, and systemic exposure. This omission was primarily due to resource constraints in the current single-center study. Future trials should include such biochemical assessments to better elucidate the relationship between niacinamide intake, circulating levels, and clinical outcomes in glaucoma.

Dietary intake of niacin was not assessed in this study, although diet can significantly influence circulating vitamin B3 levels and individual responses to supplementation. Our focus was on standardized niacinamide administration and within-patient change; however, future studies should include dietary assessments to better account for this potential source of variability.

## 5. Conclusions

Overall, niacinamide supplementation was associated with significant improvements in vision-related quality of life in glaucoma patients, particularly in areas of central and peripheral vision, as well as glare and light adaptation. These findings provide support for the neuroprotective potential of niacinamide and its impact not only on structural and physiological parameters—as suggested in previous studies—but also on subjective, patient-centered outcomes.

Future randomized controlled trials with placebo-controlled designs, larger samples and longer follow-up are needed to validate these findings and explore objective correlates such as visual field progression, contrast sensitivity, and retinal imaging.

In conclusion, this study highlights that oral niacinamide may offer a promising supportive approach in glaucoma management, with the potential to improve patients’ perceived visual capabilities and overall quality of life. The improvement in overall QoL reinforces the potential role of niacinamide as an adjunctive therapeutic strategy.

## Figures and Tables

**Table 1:** Average scores on GQL-15 baseline.

GQL-15 Items	Overall Group (*n* = 58)	Early Defect (*n* = 30)	Moderate Defect (*n* = 11)	Advanced Defect (*n* = 7)	Severe Defect (*n* = 7)
1.Reading newspapers	2.02 ± 1.05	1.9 ± 0.96	2.09 ± 1.04	2.43 ± 1.13	2.29 ± 1.5
2. Walking after dark	2.28 ± 1.27	2.2 ± 1.27	2.45 ± 1.21	2.57 ± 1.27	2.43 ± 1.62
3. Seeing at night	2.21 ± 1.15	2.23 ± 1.14	2.18 ± 1.17	2.14 ± 1.07	2.57 ± 1.51
4. Walking on uneven ground	1.93 ± 1.09	1.77 ± 0.86	2 ± 1.18	2.57 ± 1.51	2 ± 1.53
5. Adjusting to bright lights	2.41 ± 1.08	2.4 ± 1.0	2.64 ± 1.21	2.71 ± 1.25	2.29 ± 1.11
6. Adjusting to dim lights	2.43 ± 1.08	2.47 ± 1.01	2.64 ± 1.21	2.57 ± 1.27	2.29 ± 1.11
7. Going from light to dark or vice versa	2.38 ± 1.06	2.27 ± 0.91	2.64 ± 1.21	2.71 ± 1.5	2.43 ± 1.13
8. Tripping over objects	1.71 ± 0.75	1.6 ± 0.67	1.82 ± 0.6	2.14 ± 1.07	1.86 ± 0.9
9. Seeing objects coming from the side	1.58 ± 0.77	1.57 ± 0.57	1.55 ± 0.69	1.57 ± 0.98	2 ± 1.41
10. Crossing the road	1.47 ± 0.84	1.27 ± 0.52	1.45 ± 0.69	2 ± 1.29	2 ± 1.41
11. Walking on steps/stairs	1.62 ± 0.91	1.47 ± 0.63	1.63 ± 0.81	2.14 ± 1.46	2 ± 1.41
12. Bumping into objects	1.52 ± 0.73	1.4 ± 0.56	1.55 ± 0.82	1.86 ± 0.9	1.86 ± 1.07
13. Judging distance of foot to step/curb	1.67 ± 0.85	1.63 ± 0.72	1.45 ± 0.69	2.14 ± 1.21	1.86 ± 1.21
14. Finding dropped objects	1.55 ± 0.9	1.47 ± 0.68	1.45 ± 0.93	1.86 ± 1.21	2 ± 1.41
15. Recognizing faces	1.4 ± 0.75	1.33 ± 0.55	1.18 ± 0.4	1.57 ± 0.98	1.86 ± 1.46
GQL-15 subscales					
Central and near vision	17.67 ± 19.59	15.42 ± 15.29	15.91 ± 14.89	25 ± 23.04	26.79 ± 36.39
Peripheral vision	16.81 ± 18.02	14.31 ± 13.16	16.67 ± 15.25	26.78 ± 26.44	23.21 ± 29.64
Glare and dark adaptation	30.24 ± 23.12	29.31 ± 21.59	33.33 ± 23.57	35.72 ± 25.56	33.33 ± 29.95
Outdoor mobility	11.64 ± 21.06	6.67 ± 13.02	11.36 ± 17.19	25 ± 32.27	25 ± 35.36
Total GQL-15 score	28.17 ± 11.53	26.97 ± 9.21	28.73 ± 10.63	33 ± 15.18	31.71 ± 18.28

GLQ-15, Glaucoma Quality of Life-15 Questionnaire.

## Data Availability

The datasets generated during and/or analyzed during the current study are available from the corresponding author on reasonable request.

## Abbreviations

The following abbreviations are used in this manuscript:

CNV	Central and near vision
GDA	Glare and dark adaptation
GQL-15	Glaucoma Quality of Life-15 Questionnaire
IOP	Intraocular pressure
OM	Outdoor mobility
PV	Peripheral vision
QoL	Quality of life
VF	Visual field
